# Predicting the firing phase of an oscillatory neuron from its impedance profile

**DOI:** 10.1186/1471-2202-14-S1-P132

**Published:** 2013-07-08

**Authors:** Farzan Nadim, Horacio G Rotstein, David Fox

**Affiliations:** 1 Department of Biological Sciences, NJIT-Rutgers University, Newark, NJ 07102, USA; 2 Department of Mathematical Sciences, NJIT, Newark, NJ 07102, USA

## 

The activity phase of a neuron in an oscillatory network often determines what the neuron codes [1,2]. We are interested in understanding the effect of subthreshold factors that influence this activity phase. Here we develop a first-order approximation of the activity phase of a neuron receiving oscillatory input using its subthreshold impedance profile.

A neuron's subthreshold membrane potential response to sinusoidal current input with frequency *f *is sinusoidal (to first order) with amplitude and phase-shift approximated by the impedance value at *f*:$Z_{f}=∥Z_{f}∥e^{i\varphi_{f}}$. If a neuron receives suprathreshold oscillatory input at frequency *f*, the resulting change in membrane potential can be approximated with a similar amplitude and phase-shift up to the time point *t_spike _*where spike threshold is reached. This results in the following simple equation: $V_{m}\left(t_{spike}\right)=V_{rest}+A_{in}∥Z_{f}∥\text{sin}\left(2\pi f\cdot t_{spike}+\varphi_{f}\right)=V_{thresh}$ where *A_in _*is the amplitude of the input current. Assuming the reference time point of *t*_0 _= 0 in the cycle, the spike phase can be approximated as $\varphi_{spike}=f\cdot t_{spike}=\frac{1}{2\pi }\left(\text{arcsin}\frac{V_{thresh}-\;V_{rest}}{A_{in}∥Z_{f}∥}-\varphi_{f}\right)$. This approximation is valid so long as the argument of arcsin is <1 in absolute value.

As proof of principle, we used the impedance profile of a model neuron exhibiting subthreshold resonance to approximate the spike phase with a given preset spike threshold (Figure A). We also used this approximation to predict the phase of the first spike in bursting PY neurons in the crab pyloric CPG, when synaptically isolated and subjected to a sinusoidal current input at different frequencies (0.1-4 Hz; see [3]for method). The PY impedance was measured from its subthreshold response.

To our knowledge this method, despite its simplicity, has not been previously used for the approximation of spike phase using the impedance profile. The usefulness of this approximation is that changes in membrane properties due to network activity or neuromodulation can be readily measured in the impedance profile and this knowledge can be used to predict how the neuron changes its response phase during network activity.

**Figure 1:**
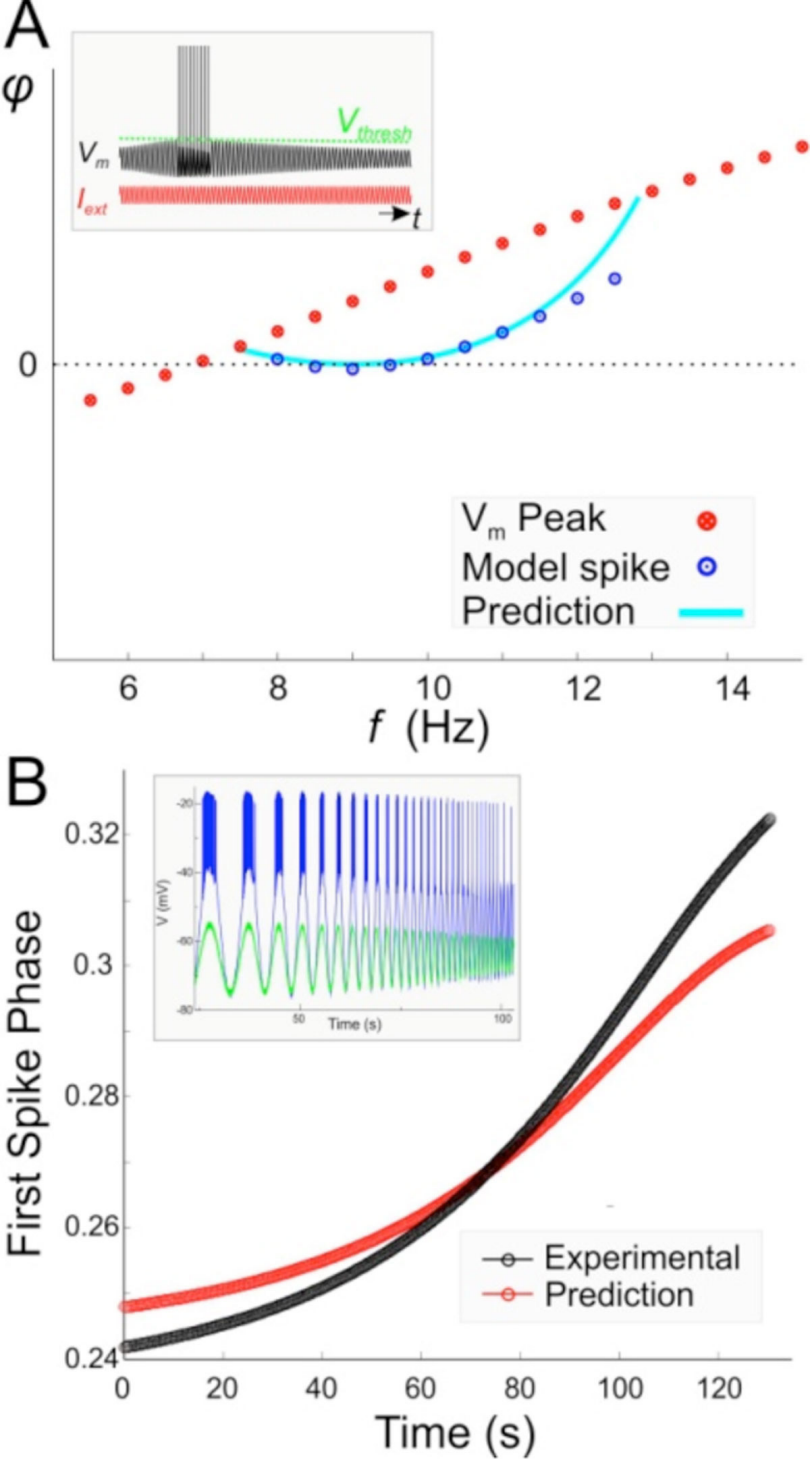
**A. The spike phase of a resonate-and-fire model predicted from the impedance profile**. B. The spike phase of the PY neuron subject to a ZAP input (inset) predicted from its subthreshold response (green).

There are three sources of error in this approximation. First, the membrane impedance in biological neurons is nonlinear and does not scale linearly with the amplitude of the input current, especially when the neuron transitions from sub- to suprathreshold activity. However, the shift in membrane impedance can be tracked in both model and biological neurons. Second, spike threshold is dependent on input frequency. This dependence can also be tracked. Third, because of nonlinearities, the membrane potential response of a neuron is not perfectly sinusoidal. As seen in Figure 1B, despite these (nonlinear) sources of error, our method can provide a good approximation of spike phase. We are in the process of estimating spike phase in response to synaptic inputs arriving at a fixed frequency.
